# Perspectives and practices of nutritionists on dietary supplements for elite soccer teams: a cross-sectional survey study

**DOI:** 10.3389/fspor.2023.1230969

**Published:** 2023-08-11

**Authors:** Rodrigo Abreu, Catarina B. Oliveira, João Brito, Vitor H. Teixeira

**Affiliations:** ^1^ Portugal Football School, Portuguese Football Federation, FPF, Oeiras, Portugal; ^2^ Faculty of Nutrition and Food Sciences, University of Porto (FCNAUP), Porto, Portugal; ^3^ CHRC, NOVA Medical School, Faculdade de Ciências Médicas, NMS, FCM, Universidade NOVA de Lisboa, Lisboa, Portugal; ^4^ Research Centre in Physical Activity, Health, and Leisure, CIAFEL, Faculty of Sport, University of Porto, Porto, Portugal; ^5^ Laboratory for Integrative and Translational Research in Population Health (ITR), Porto, Portugal; ^6^ Futebol Clube do Porto SAD, Porto, Portugal

**Keywords:** football, questionnaire, ergogenic, recovery, nutrition knowledge

## Abstract

**Introduction and objectives:**

Dietary supplements are part of the nutritional strategies frequently applied in sports performance support. With growing research on this subject and high demand from athletes, nutritionists need to keep up to date with the latest evidence and utility of dietary supplements, particularly in real-world contexts. As information about the use of dietary supplements among elite soccer players is still scarce, this work aimed to know how nutritionists working with elite soccer teams perceive and use these substances in their daily practice.

**Methods:**

A questionnaire previously used to describe nutritionists’ beliefs and attitudes regarding the use of dietary supplements in a clinical context was adapted for this study. The online questionnaire was addressed to nutritionists working with elite soccer teams from six European Leagues and Brazil, between November 2022 and February 2023.

**Results:**

Overall, the participants considered themselves well-trained (76.9%), knowledgeable (95.4%), and interested in dietary supplements (95.4%). The majority (70.8%) of the participants agreed or strongly agreed to recommend dietary supplements to soccer players. Personal usage of dietary supplements was associated with recommending supplements (*p* < 0.001), but no relationships were found with years of experience and academic level.

**Discussion:**

Nutritionists working with elite soccer players consider the use of dietary supplements for performance-enhancement purposes and not only to compensate for nutritional deficits, which might contribute to their higher interest, training and perceived knowledge about this topic. Participants recognize players’ interest in dietary supplements, and are mindful of the safety and efficacy of these products. The present study suggests that nutritionists working with elite soccer teams are among the highest prescribers of dietary supplements, although personal usage is lower than that of nutritionists working in a clinical context.

## Highlights

• Knowledge about the perspectives and practices of nutritionists working with elite soccer teams are important to better understand the reasons for recommendation and use of dietary supplements.
• Most nutritionists in this survey recommend dietary supplements to elite soccer players, and are aware of the challenges regarding safety and effectiveness.
• Nutritionists working with elite soccer teams are interested in dietary supplements and also recognize the importance of training and research in this area.

## 1. Introduction

Nutrition care is well established as part of support strategies used to improve performance and recovery in soccer (1). Nevertheless, the way nutritional strategies are applied can change considerably depending on numerous factors, such as players’ individual preferences or team competitive calendars, and has deserved the attention of researchers and practitioners in recent years. Energy and nutrient requirements (2, 3) and the effects of dietary supplements are some of the topics studied in the context of elite soccer. Available data suggest a high prevalence of the use of dietary supplements by elite soccer players (4, 5). Given the growing number of research enrolling dietary supplements and the high number and variety of products available on the market, it is relevant to understand better how these substances are being used in soccer.

Several definitions exist for dietary supplements, including those established by regulatory entities [such as EFSA in the European Union (6)]. However, for the purpose of the present study, the definition recently proposed in the International Olympic Committee (IOC) consensus statement (“a food, food component, nutrient, or non-food compound that is purposefully ingested in addition to the habitually-consumed diet with the aim of achieving a specific health and/or performance benefit”) was considered the most suitable (7). This includes substances such as caffeine, creatine, or vitamins, and products like isotonic drinks or protein shakes, typically used among soccer players (8). Recent publications have described the evidence regarding the benefits of dietary supplements in soccer players’ performance or recovery (1, 9). However, information on how nutritionists address the use of these products remains scarce.

To our knowledge, the first work addressing the practices and recommendations of sports nutritionists was conducted by Grandjean in 1993 (10). In this survey, it was found a positive correlation (*p *< 0.008) between personal usage of dietary supplements and the recommendation of these products to athletes. Nevertheless, most of the professionals worked with recreational athletes and were more concerned about encouraging a healthy diet than improving athletic performance. More recently, other surveys assessed nutrition knowledge, practices and perceptions of nutritionists regarding dietary supplements (11–14), but research was focused on a clinical context, and the use of dietary supplements to balance food intake or address particular health conditions. In the context of sports nutrition, Wardenaar and Hoogervorst (15), in a survey conducted among sports health professionals (18% of which were nutritionists) working with Olympic and non-Olympic athletes, noted that sports dietitians were ranked as the most knowledgeable professionals about nutritional supplements (74%). Not surprisingly, nutritionists have been considered among the most well-informed professionals and preferred source of information about dietary supplements in surveys conducted among athletes (16). Thus, it is important to better understand how nutritionists’ perspectives on dietary supplements can influence their use by athletes, particularly at the elite level.

In elite soccer, studies assessing the use of dietary supplements among players are still scarce (4, 5, 8) and the available results pointed out health concerns as the main reason for using these products. Yet, accelerate recovery, improve performance, and prevent injury are also among the most frequently reported motivations for using dietary supplements. These data suggest that male and female elite players consider dietary supplements as part of the strategies for dealing with physical and physiological demands of elite soccer. Though, since the training and playing demands have increased (17), support teams, namely health and performance professionals, are more required to attend to the needs imposed by such demanding fixtures properly. The high number of matches per season [nowadays, some players from the most competitive teams can play over 60 games per season (18)] and the popularity of soccer also mean more visibility and scrutiny of the practices and procedures of players and teams. Additionally, as elite teams and players are considered the benchmark of good practices for all soccer players, nutritionists working in the context of elite sports must be able to ensure evidence-based nutritional strategies are adjusted to the very specific conditions and needs of the real world. Thus, the present study aimed to assess the perceptions and practices of nutritionists supporting elite soccer teams regarding dietary supplements.

## 2. Materials and methods

### 2.1. Participants and settings

The scope of this work was limited to nutritionists working with soccer players from elite clubs. A list of the nutritionists working with elite soccer teams from six European leagues (English Premier League, Spanish La Liga, Italian Serie A, German Bundesliga, French Ligue 1, and Portuguese Primeira Liga) and Brazil (Serie A) was defined, based on authorś personal knowledge, as well as information available from each club. After approval by the Ethical Committee of the Portugal Football School (PFS 16/2022), the nutritionists were contacted via e-mail and professional and personal networks. Invitations to participate were also addressed to all clubs via institutional e-mail.

Data collection took place between November 7th 2022 and February 17th 2023 using an anonymous questionnaire (available in Supplementary Appendix S1), adapted from a study used for a similar purpose (12) after obtaining the authors’ permission. The questionnaire was provided in English and administered online using Microsoft Forms®.

### 2.2. Variables and instruments

The questionnaire used in the present study comprised 19 questions grouped into three sections: (a) participants’ information; (b) perceptions about dietary supplements; and (c) practices regarding the use and recommendation of dietary supplements. Overall, questions from the original questionnaire used by Marx et al. (12) were kept, but since it was focused on nutritionists working within a clinical context, some minor adaptations were applied in order to fit the nutritional context of elite soccer. Generally, questions using the word “patients” were adapted to “soccer players” and questions about the perceptions and recommendation of dietary supplements were adapted to include answers regarding the conditions faced by sports nutritionists (such as, athletic performance enhancement or fatigue recovery).

#### 2.2.1. Information from participants

Information from participants was collected via open-ended and multiple-choice questions. Data collected in this section included the following: age; contractual relationship with the club; experience, measured as years working with nutrition in a soccer club context; education, measured as completed degree; and workload with soccer players.

#### 2.2.2. Perceptions about dietary supplements

In this section, several perceptions about dietary supplements were assessed using a list of 20 sentences, to which participants were asked to respond using a 5-point Likert scale (from 1, strongly disagree, to 5, strongly agree). Questions included statements regarding participants perception about their personal interest, training, and knowledge in dietary supplements, about nutritionists’ role in research, education and prescription of dietary supplements, and also about dietary supplements use in soccer.

#### 2.2.3. Practices regarding the use and recommendation of dietary supplements

Finally, the use and recommendation of dietary supplements was also assessed with multiple-choice questions. Participants were asked if they personally used dietary supplements in the past 6 months, and if they sell dietary supplements as part of their practice. Recommendation of dietary supplements, as well as barriers and enablers for recommending were also assessed in this section.

### 2.3. Statistical methods

Statistical analysis was performed using Jamovi 2.3.21.0 software. Normal distribution was tested using the Shapiro-Wilk test. Descriptive statistics were reported as percentages, as mean (standard deviation) for variables following a normal distribution (i.e., age), or median (Q_1_; Q_3_) for variables not following a normal distribution (i.e., participants’ experience). The items of the 5-point Likert-type scales were grouped into three categories for the analysis (strongly disagree and disagree; neutral; agree and strongly agree). The Mann-Whitney test was used to compare the difference between the experience (continuous variable, not normally distributed) of participants selling and not selling dietary supplements (nominal variable, dichotomous). As Likert-type scales (e.g., recommending dietary supplements) can be regarded as both nominal and ordinal variables, the following analysis were conducted. The Kruskal-Wallis test was used to compare the difference between recommending dietary supplements (nominal variable, three independent groups) with regard to participants’ experience (continuous variable, not normally distributed). Spearman's correlation was used to assess the relationship between recommending dietary supplements (ordinal variable) and the participants’ education (ordinal variable) and participants’ experience (continuous variable, not normally distributed). The Fisher's exact test was used to assess the association between selling (nominal variable, dichotomous) and taking dietary supplements (nominal variable, dichotomous). Finally, the Freeman-Halton extension of the Fisher's exact test was used to assess the association between recommending dietary supplements (nominal variable, three independent groups) and selling dietary supplements (nominal variable, dichotomous) and between recommending and taking dietary supplements (nominal variable, dichotomous). Values of *p* < 0.05 were considered statistically significant.

## 3. Results

**Figure 1:**
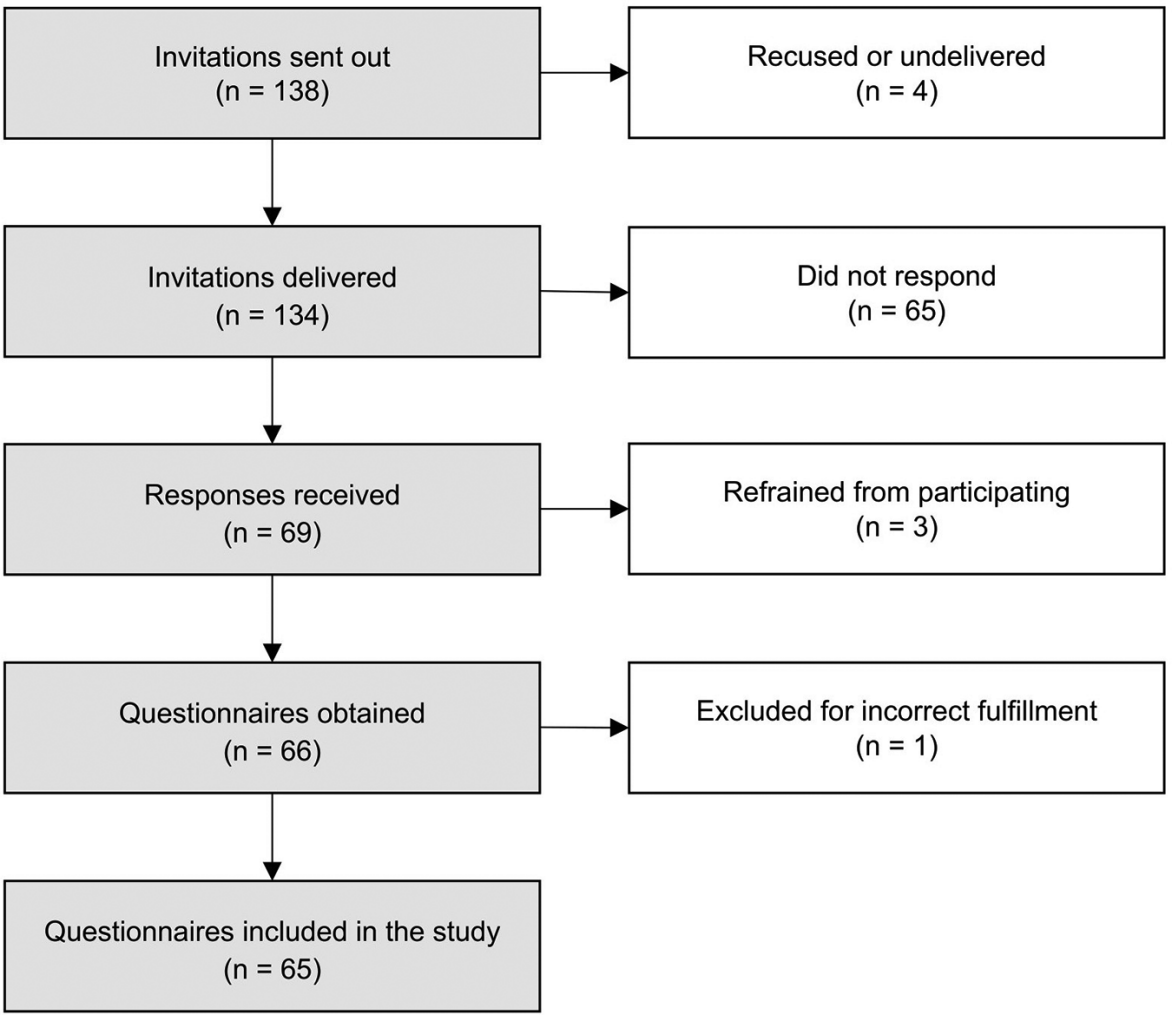
Flow diagram of participant recruitment.

One hundred thirty-eight invitations were sent out, with four recused or undelivered. From those, 69 responses were received (50.0% response rate); however, three belonged to nutritionists who refrained from participating due to their club's policy. Of the 66 questionnaires obtained, one was excluded due to incorrect fulfillment. Therefore, 65 participants were included in the statistical analysis presented (Figure 1).

A resume of participants’ characteristics can be found in Table 1.

**Table 1:** Participants’ characteristics.

Age	34.7 (5.6) years
Years as a nutritionist in soccer	6.0 (4.0; 9.3) years
Education	
Diploma	9.2%
Bachelor	26.2%
Masters	50.8%
PhD	13.8%
Contractual relationship with soccer club	
Full-time	60.0%
Part-time	32.3%
Consultant/advisor	7.7%
Workload spent with soccer players	
Daily	72.3%
At least once a week	26.2%
Occasionally (every 2 weeks, once a month, beginning of season, upon request)	1.5%

Values are expressed as percentages, mean (standard deviation), or median (Q_1_; Q_3_). Data were available for all participants (*n* = 65) except for age (*n* = 63) and years as a nutritionist in soccer (*n* = 64).

### 3.1. Perceptions regarding dietary supplements

**Figure 2:**
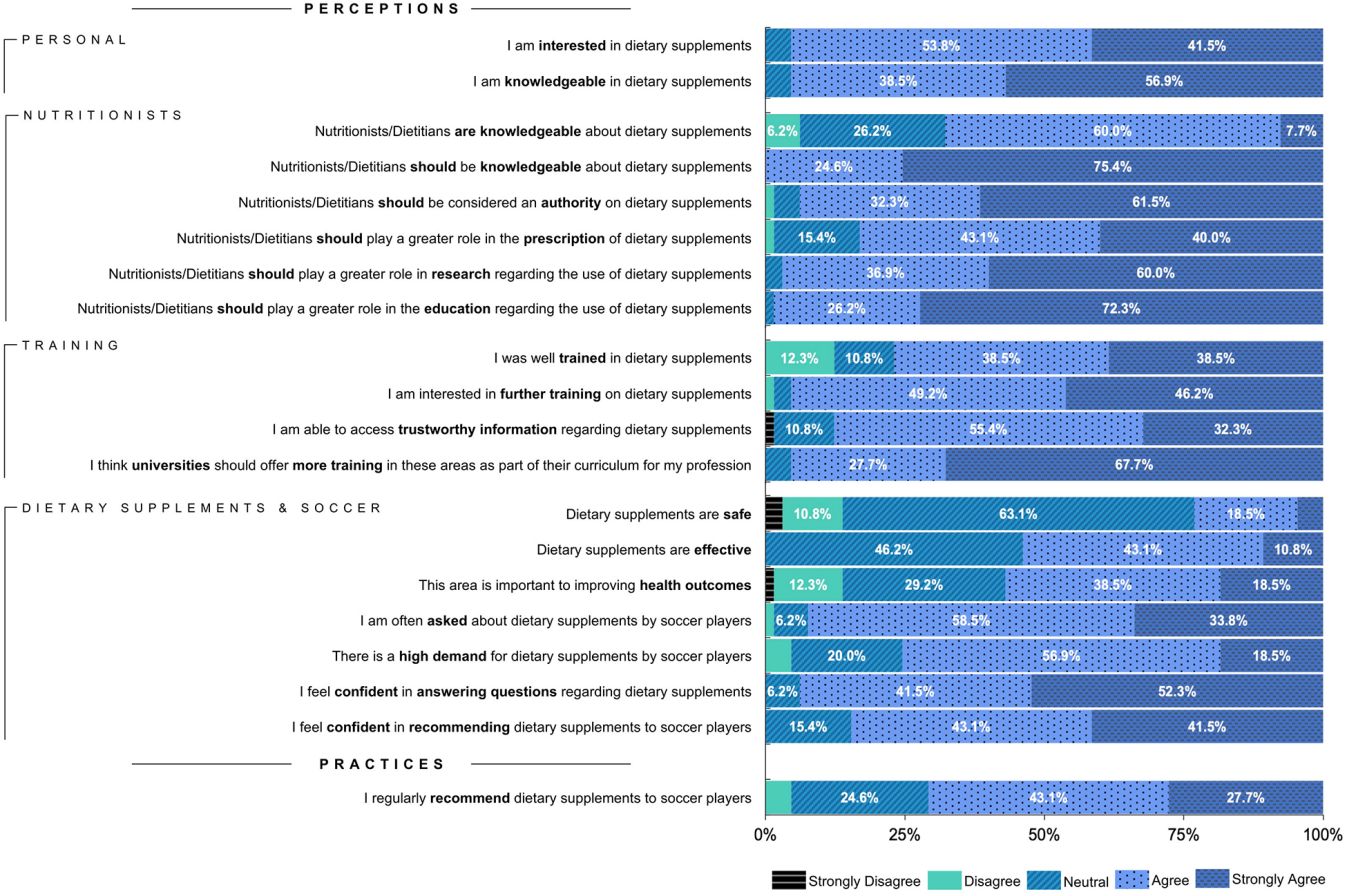
Nutritionists’ perceptions regarding dietary supplements.

Results from the questions assessing nutritionists’ perceptions regarding dietary supplements using a 5-point Likert-type scale are summarized in Figure 2. Almost all participants reported interest in dietary supplements (95.4% agreed or strongly agreed with this statement) and considered themselves knowledgeable about supplementation (95.4%). When asked if they were well trained in dietary supplements, 76.9% of participants agreed or strongly agreed, and 87.7% agreed or strongly agreed that they can access trustworthy information about dietary supplements.

Concerning participants’ perceptions about their professional colleagues, most believe that nutritionists/dietitians are knowledgeable about dietary supplements (67.7% agreed or strongly agreed with this statement) and that nutritionists/dietitians should be considered an authority on dietary supplements (93.8%). Additionally, most participants agreed or strongly agreed that nutritionists/dietitians should play a more significant role in the prescription of dietary supplements (83.1%), education regarding dietary supplements (98.5%), and research on the use of dietary supplements (96.9%).

Regarding participants’ perception of the efficacy and safety of dietary supplements, approximately half believe dietary supplements are effective (53.8% agreed or strongly agreed with this statement); however, only 23.1% agreed or strongly agreed that dietary supplements are safe.

When asked who the primary source of information on dietary supplements should be, almost all participants pointed out nutritionists/dietitians (98.5%), followed by medical doctors (55.4%), pharmacists (24.6%), and sports scientists (24.6%). Conversely, when participants were asked who the primary source of information was, the most frequent responses were nutritionists/dietitians (73.8%), coach/fitness coaches (67.7%), and friends and family (53.8%).

Sports performance (physical and physiological) (93.8%), fatigue recovery (81.5%), and sports performance (mental and cognitive) (76.9%) were mentioned as the areas where dietary supplements are most effective.

Most participants agreed that they are often asked about dietary supplements (92.3% agreed or strongly agreed with this statement) and recognized a high demand for dietary supplements by soccer players (75.4% agreed or strongly agreed with this statement).

Finally, participants considered that the topics they would like to learn more about were the usage of dietary supplements for sports performance (69.2%), specific dietary supplements (53.8%), and reliable sources of information (30.8%).

### 3.2. Practices regarding dietary supplements

Of all participants, 15.4% admitted selling dietary supplements, with all of them considering there is no conflict of interest in such practice. When asked about their personal use of dietary supplements, 58.8% reported taking at least one in the last 6 months.

Participants reported getting information regarding dietary supplements mainly from evidence databases and academic journals (93.8%), followed by conferences (58.5%), and guidelines by their professional bodies (43.1%). Regarding the minimum level of evidence required before feeling confident in utilizing or recommending specific dietary supplements to soccer players, most participants reported systematic reviews (44.6%), randomized controlled trials (36.9%), and meta-analysis (7.7%).

Although 26.2% of participants reported not perceiving barriers to recommending dietary supplements to soccer players, concerns about the regulation of dietary supplements (50.8%), potential adverse effects of dietary supplements (26.2%), and perceived lack of efficacy and quality of dietary supplements (both with 24.6% of responses), were the most frequent answers. On the other hand, enablers to recommending the use of dietary supplements to soccer players included sufficient training (78.5%), sufficient autonomy to recommend dietary supplements (63.1%), and sufficient research to show the efficacy, as well as high-quality dietary supplements available on the market (both options with 47.7% of responses).

Regarding the factors that might relate to the recommendation of dietary supplements, relationships between individual factors (i.e., personal use, selling, experience, and education) and advice on dietary supplements were investigated. No differences were found between the median years of experience of participants who sell and those who do not sell dietary supplements (*p* = 0.656). In addition, the median years of experience did not differ with regard to recommending dietary supplements (*p* = 0.225). An association between personal usage of dietary supplements and recommending dietary supplements to soccer players was found (*p* < 0.001). However, no association was found between selling dietary supplements and recommending their use (*p* = 0.123), nor between selling supplements and use them personally (*p* = 0.175). Finally, no relationships between nutritionists’ experience (*p* = 0.131) or education (diploma, bachelor, master, or Ph.D.) (*p* = 0.861) and recommending dietary supplements were found.

## 4. Discussion

In general, nutritionists working with Europe and Brazil's top soccer teams demonstrate a high interest in dietary supplements and consider themselves knowledgeable about them. Most participants on this survey regularly recommend supplements to elite soccer players, and most also personally use them.

To the best of our knowledge, this is the first time that the practices and perceptions of nutritionists working in the context of elite soccer teams regarding dietary supplements have been explored. In 1993, Grandjean (10) assessed sports nutritionists’ practices, including recommendations regarding dietary supplements, among others. More recently, similar works have assessed the beliefs and attitudes towards dietary supplements of nutritionists working in a clinical context (11–13). Notably, differences in some of the objectives and methodology between studies make it difficult to compare results. Even so, topics such as interest, perceived knowledge, and recommendation of dietary supplements, as well as personal usage by nutritionists, were assessed in these studies.

In our work, 95.4% of the nutritionists agreed or strongly agreed that they are interested in dietary supplements, a higher percentage than that found in previous studies: in 2006, Hetherwick et al. (11) found that 87% of registered dietitians in the USA were interested in dietary supplements, and, in 2016, Marx et al. (12) reported that 68% of dietitians in Australia agreed or strongly agreed that they are interested in dietary supplements.

When comparing the perceived knowledge about dietary supplements, we can also observe differences between nutritionists working with elite soccer teams (95.4% agreed or strongly agreed that they are knowledgeable about dietary supplements, according to our findings) and those working in the clinical context [60% agreed or strongly agreed that they are knowledgeable about dietary supplements, in the survey by Marx et al. (12)]. More recently, a survey conducted among licensed dietitians working in a clinical context in Lebanon (13) assessed knowledge about dietary supplements, founding that 30% had a good knowledge score and 46% had a very good knowledge score.

Regarding their practices, 70.8% of nutritionists working with elite soccer teams agreed or strongly agreed that they regularly recommend dietary supplements. These results compare similarly with the ones found among registered dietitians in California (74% of participants declared to recommend dietary supplements) (13), but are higher than those reported by more recent works: only 27% of dietitians in Australia agreed or strongly agreed that they regularly recommend dietary supplements (12). And in 2021, 39.5% of licensed dietitians in Lebanon reported recommending dietary supplements to their patients (13).

Interestingly, in our study, the percentage of nutritionists declaring to use dietary supplements personally (58.8%) was lower when compared to the results from the surveys carried out with professionals working in a clinical context; 65% of dietitians in Australia (12), 69% of dietitians in California (11) and 73.7% of dietitians in Lebanon (13) declared to use dietary supplements. In the survey conducted by Grandjean (10), 55.0% of the sports nutritionists declared using dietary supplements. On the contrary, we found a higher percentage (15.4%) of nutritionists claiming to sell dietary supplements compared to 4% reported by Marx et al. (12) and 5.3% in the work of Nacouzi et al. (13).

The specific context of sports nutrition, where dietary supplements can be used for performance-enhancement purposes and not only to compensate for nutritional deficits or, allegedly, prevent diseases, might explain some of the differences between the results observed in our survey and those conducted among dietitians working in a clinical context. Dietary supplements such as creatine, caffeine, or protein powders currently gather broad consensus and evidence regarding the benefits for athletes’ physical performance and body composition management (1, 7, 19–21), meaning that nutritionists working with elite soccer players may be more likely to recommend these substances. Also, the health and performance demands of elite soccer might also explain why nearly all nutritionists who participated in our survey considered themselves interested and knowledgeable about dietary supplements, more than their fellows working in a clinical context (11–13). These results should be interpreted with caution as these variations might also be related to differences in cultural habits, purchase power, and availability of dietary supplements in each country, as studies were conducted in the United States of America (specifically in California), Australia, Lebanon, and, in our study, in six different European countries and Brazil. It is also worth comparing the results from our survey with the available data from elite players’ responses to questionnaires about the usage of dietary supplements. Although 70.8% of the nutritionists inquired agreed or strongly agreed to regularly recommended dietary supplements, reported use of these substances by elite players ranges between 82.0% (8) and 98.2% (5). This might suggest that players can be using dietary supplements by themselves or recommended by others than the nutritionists from their clubs. But it should be mentioned that the available studies regarding the usage of dietary supplements by elite players are from Turkey (5), Saudi Arabia (4) or female National Teams (8), whilst our survey was conducted with nutritionists from six elite European Leagues and Brazil.

Additionally, it would be worth understanding if the dietary supplements reportedly used by elite players coincide with those recommended by nutritionists and with established evidence regarding their benefits. Although the questionnaire used did not assess which dietary supplements participants recommend to players, sports drinks, vitamins (vitamin C, vitamin D, and multivitamins) and minerals (namely, magnesium) have been the most frequently reported by soccer players in previous studies (4, 5, 8). Participants in our survey recognize sports performance (athletic and cognitive) and fatigue recovery as the areas where dietary supplements can be most effective, which is supported by evidence from recent systematic reviews with professional and elite soccer players (22, 23). Even if it is not possible to directly compare results from available surveys conducted with players and nutritionists, it would be relevant to understand if there are differences between practitioners’ recommendations and dietary supplements used by players, as this raises efficacy and safety questions. Therefore, future research should address these issues, either through validated tools for the specific context of elite soccer, or through the application of questionnaires to nutritionists and players supported by them. We believe this will allow a better understanding of how nutritionists’ perceptions and practices impact elite soccer players’ dietary supplement usage and, consequently, their overall performance.

### 4.1. Strengths and limitations

To our knowledge, this is the first work about the perceptions and practices of nutritionists on dietary supplements for elite soccer players. Research with elite athletes is typically more scarce, either because these athletes are fewer, or because their availability to participate in studies is also more limited. Given the role of nutritionists working in the context of elite soccer in the definition and application of nutritional strategies, this survey allows a first portrait of the opinion of these professionals and their recommendations. The results of this work clearly show that nutritionists working with major leagues clubs are interested and dedicated to knowing more about dietary supplements and their applications in the specific context of elite soccer. Additionally, the application of instruments already used in previous surveys, contributes to a better understanding of the perspectives of these nutritionists, when compared with colleagues who work in a clinical context.

Even so, the present survey has some limitations to consider in future research. Given that the questionnaire used in this work was adapted from one designed and validated for a clinical context, it should be considered the development of a specific questionnaire for professionals working in sports nutrition. This questionnaire may include not only the perceptions, but also directly assess some nutritional knowledge. Finally, it will also be relevant that the tools to be developed for this purpose include questions about which supplements are recommended by nutritionists and reasons for doing so.

## 5. Conclusions

Current evidence about the benefits of dietary supplements on athletic performance and recovery, paired with the great interest shown by players, are important reasons for knowing perceptions and practices of nutritionists working with elite soccer teams. For the first time, these practitioners’ perspectives were explored, resulting in better knowledge on their opinion about dietary supplements and their recommendation to elite soccer players. Next steps in the research of this topic should contribute to understand how the nutritionists’ recommendations are followed by players.

## Data Availability

The original contributions presented in the study are included in the article/**Supplementary Material**, further inquiries can be directed to the corresponding author.
