# Supplementary material for: Perspectives and practices of nutritionists on dietary supplements for elite soccer teams: a cross-sectional survey study
Source: Front Sports Act Living. 2023 Aug 11;5:1230969. doi: 10.3389/fspor.2023.1230969 (PMC10450918; doi:10.3389/fspor.2023.1230969)
Supplement: Supplementary file 1 [file Datasheet1.docx]

1. **INTRODUCTION**

**Background**

The aim of this survey is to explore the views, behaviours, and attitudes of nutritionists/dietitians in regards to the use of dietary supplements.

**Is this you?**

This survey is aimed specifically towards nutritionists/dietitians who currently work with nutritional support to soccer players in Clubs.

**Please read the following**

To help guide your responses to this survey, please use the following definition when thinking about the term “dietary supplements”:

A food, food component, nutrient, or non-food compound that is purposefully ingested in addition to the habitually consumed diet with the aim of achieving a specific health and/or performance benefit.

For the sake of this study, “dietary supplements” DOES NOT include:

WADA banned substances and drugs that need to be prescribed by the Medical Doctor.

**Ethics information**

The study is voluntary and anonymous. We will collect non-identifiable data only and demographic data will be pooled. We anticipate this survey will take approximately 10-15 minutes to complete. This research protocol has been approved by the Portugal Football School Ethics Committee (PFS 16/2022). If you have any questions regarding this project, please contact the principal researcher:

Rodrigo Abreu

[rodrigo.abreu@fpf.pt](mailto:rodrigo.abreu@fpf.pt)

(+351) 919 514 735

Please click the button below if you give your consent to participate in this study.

Yes

No

1. **DEMOGRAPHIC INFORMATION**

2.1 What is your age?

2.2 What kind of contractual relationship do you have with the club?

- Full time

- Part-time

- Consultant

- Other (please specify)

2.3 How many years have you been working with nutrition in a soccer club context?

2.4 What is the highest level of education you have completed?

- Diploma

- Bachelor

- Masters

- PhD

2.5 Approximately how much of your workload is spent with soccer players?

- Daily

- At least once a week

- Occasionally (every two weeks, once a month, beginning of season, upon request)

- Never (external advisor, remote support)

2.6 Do you sell supplements as part of your clinical practice?

- Yes

- No

2.7 Do you consider this a conflict of interest?

- Yes

- No

2.8 Do you feel you have any other potential conflicts of interest that may bias your response to this survey? (For example, profit gain from advertising specific supplements or Club sponsoring)

- Yes

- No

1. **ATTITUDES, BARRIERS, AND BELIEFS**

3.1 In a scale from 1 (strongly disagree) to 5 (strongly agree), please describe how much you agree or disagree with the following statements regarding dietary supplements.

3.1.1 I am knowledgeable about dietary supplements.

3.1.2 I am interested in dietary supplements.

3.1.3 Nutritionists/Dietitians are knowledgeable about dietary supplements this area.

3.1.4 I was well trained in dietary supplements.

3.1.5 This area is important to improving health outcomes.

3.1.6 Dietary supplements are effective.

3.1.7 Nutritionists/Dietitians should be knowledgeable about dietary supplements.

3.1.8 Nutritionists/Dietitians should be considered an authority on dietary supplements.

3.1.9 There is a high demand for dietary supplements by soccer players.

3.1.10 I am often asked about dietary supplements by soccer players.

3.1.11 I feel confident in answering questions regarding dietary supplements.

3.1.12 I feel confident in recommending dietary supplements to soccer players.

3.1.13 I am interested in further training on dietary supplements.

3.1.14 Dietary supplements are safe.

3.1.15 Nutritionists/Dietitians should play a greater role in the prescription of dietary supplements.

3.1.16 Nutritionists/Dietitians should play a greater role in the education regarding the use of dietary supplements.

3.1.17 Nutritionists/Dietitians should play a greater role in research regarding the use of dietary supplements.

3.1.18 I think universities should offer more training in these areas as part of their curriculum for my profession.

3.1.19 I am able to access trustworthy information regarding dietary supplements.

3.1.20 I regularly recommend dietary supplements to soccer players.

PERSONAL USAGE

3.2 Do you personally take (now, or on the last 6 months) one or more dietary supplement?

- Yes

- No

3.3 For soccer players, who should be the primary source(s) of information regarding dietary supplements? (Tick as many as you feel suitable)

- Doctors

- Pharmacists

- Nutritionists/Dietitians

- Nurses

- Physiotherapist

- Coach/fitness coach

- Sport scientist

- Friends and family

- Television/radio

- Internet

- Social Media

- Other

3.4 For soccer players, who do you believe are the primary source(s) of information regarding dietary supplements? (Tick as many as you feel suitable)

- Doctors

- Pharmacists

- Nutritionists/Dietitians

- Nurses

- Physiotherapist

- Coach/fitness coach

- Sport scientist

- Friends and family

- Television/radio

- Internet

- Social Media

- Other

3.5 Where do you get your information regarding dietary supplements? (Tick as many as you feel suitable)

- Conferences

- Workshops

- Colleagues

- Friends and family

- Evidence databases and Academic journals

- Guidelines by your professional body

- Television/radio

- Social Media

- Books

- Branded Materials (sales slicks, vade mecum, other information provided by brands)

- Other (please specify)

3.6 What is the minimum level of evidence that you require before you would feel confident utilising or recommending specific dietary supplements in your workplace? Please select only one response

- Cell culture and lab research

- Animal studies

- Case studies

- Observational and epidemiological studies

- Non-randomised, open label human trials

- Randomised controlled trials

- Systematic reviews

- Meta-analysis

- Evidence databases

- Other (please specify)

- Regarding these publications, what is the criteria for your choice (select only one):

a) Only with soccer players

b) With athletes in general

c) Not specified

3.7 In relation to your answer to the previous question, approximately how many of these studies/guidelines would need to be published before you utilise specific dietary supplements?

- One

- Two to four

- Five or more

- At least one systematic review/meta-analyses

3.8 What area do you think dietary supplements are most effective for? Please tick as many as you feel necessary.

- Sports performance (physical and physiological)

- Sports performance (cognitive and mental)

- Acute-care (e.g. injury rehabilitation, post-operative recovery)

- Injury prevention

- Fatigue recovery

- Weight loss

- Sleep disorders

- Mental and cognitive issues (e.g. anxiety, stress)

- Prevention of other chronic diseases (e.g. cholesterol management)

- Management and treatment of other chronic diseases (e.g. CVD, T2DM)

- Digestive disorders (e.g. IBS, Crohn's disease)

- Cancer prevention

- Dietary supplements are not effective for any area

- Other (please specify)

3.9 What do you feel are the major barriers to you recommending the use of dietary supplements to soccer players? Please tick as many as you feel necessary.

- A lack of training in this area

- A lack of confidence in this area

- Concerns regarding potential interactions with other treatments

- Concerns regarding potential negative effects of dietary supplements

- Concerns about the regulation of dietary supplements

- Perceived lack of efficacy of dietary supplements

- It may conflict with the advice of other members from the soccer players medical team

- Lack of authority to recommend dietary supplements to soccer players

- A lack of interest in this area

- Concerns regarding financial burden on player

- Perceived lack of quality dietary supplements on the market

- Other (please specify)

- No barriers, I recommend the use of dietary supplements.

3.10 What do you feel are the major enablers to you recommending the use of dietary supplements to soccer players? Please tick as many as you feel necessary.

- I have sufficient training in this area

- There are sufficient regulations regarding dietary supplements

- There is sufficient research to show the efficacy of dietary supplements

- There is sufficient research to show the safety of dietary supplements

- The physician and medical team of soccer players are supportive of the use of dietary supplements

- I have sufficient autonomy to recommend dietary supplements to soccer players

- Dietary supplements are cost-effective.

- There are high-quality dietary supplements available on the market.

- Other (please specify)

- No enablers, I do not currently recommend the use of dietary supplements.

3.11 Which area would you like to learn more about? Please tick as many as you feel suitable.

- Specific dietary supplements

- The usage of dietary supplements for sports performance

- The usage of dietary supplements for specific diseases (e.g. cancer)

- Drug-supplement interactions

- Regulatory issues regarding dietary supplements

- Reliable sources of information regarding dietary supplements

- Adverse effects of dietary supplements

- Other (please specify)

- None, I do not wish to learn about dietary supplements
